# The Importance of the Solids Loading on Confirming the Dielectric Nanosize Dependence of BaTiO_3_ Powders by Slurry Method

**DOI:** 10.1155/2013/120983

**Published:** 2013-06-06

**Authors:** Wei Zhou, Yi Mei Nie, Shu Jing Li, Hai Yan Liang

**Affiliations:** School of Science, Beijing Technology and Business University, Beijing 100048, China

## Abstract

The dielectric nanosize dependence of BaTiO_3_ powders was investigated by the slurry method, where two series of BaTiO_3_ slurries with 10 vol% and 30 vol% solids loadings were prepared as model samples. Applying the Bruggeman-Hanai equation, the high-frequency limiting permittivity (*ε*_*h*_) of the slurries was extracted from the dielectric spectra. The *ε*_*h*_ of the 10 vol% slurry showed abnormal size independence in the range from 100 nm to 700 nm, and the *ε*_*h*_ of the 30 vol% slurry exhibited good agreement with the previous prediction. Through analysing quantitatively the response of *ε*_*h*_ to the changing permittivity of the powders under different solids loading, it was found that the *ε*_*h*_ of the slurry with lower solids loading is more inclined to be interfered by the systematic and random errors. Furthermore, a high permittivity value was found in the BaTiO_3_ powders with 50 nm particle size.

## 1. Introduction

With the achievement of the particle size from several microns downward tens of nanometers, the issue about the nanosize effect of BaTiO_3_ has attracted intensive attentions [1–3]. However, the reported critical size for BaTiO_3_ powders spreads out in the wide range between 25 and 110 nm [4, 5]. The intrinsic reason is that the grain of BaTiO_3_ powders would grow up irreversibly during the high temperature sintering. So it is very difficult to obtain the exact critical size of BaTiO_3_ by means of using traditional solid-state pellet technique. Recently, the dielectric evaluation of ceramic slurries as an *in situ* method has been of primary interest [6, 7]. However, the slurry method presented that the permittivity of BaTiO_3_ powders keeps basically constant when the particle size is above 200 nm [8]. The result deviates the present theories about the size dependence of BaTiO_3_ powders and implies that some defects in the slurry method need to be improved. In this paper, we focused on the effect of the solids loading on the slurry method and offered some accurate and valuable information to the dielectric nanosize dependence of BaTiO_3_ powders.

## 2. Experimental

BaTiO_3_ powders with seven kinds of particle sizes, 50, 100, 200, 300, 400, 500, and 700 nm, were commercially obtained from Inframat Advanced Materials, USA. The purities of all powders are higher than 99.95%, and the room-temperature density is 5.85 g/cm^3^. BaTiO_3_ slurries at two solids loadings, 10 vol% and 30 vol%, were prepared by ultrasonically mixing powders with a propylene carbonate (*ε* = 66.7 at 25°C, from Fisher Scientific) for 30 min. The slurries were then placed in a liquid dielectric test fixture (Agilent Tech., 16452A), and the frequency dependence of the permittivity of the slurries was measured with an impedance analyzer (Agilent Tech. 4294A) from 40 Hz to 20 MHz at 25°C. 

## 3. Result and Discussion

**Figure 1:**
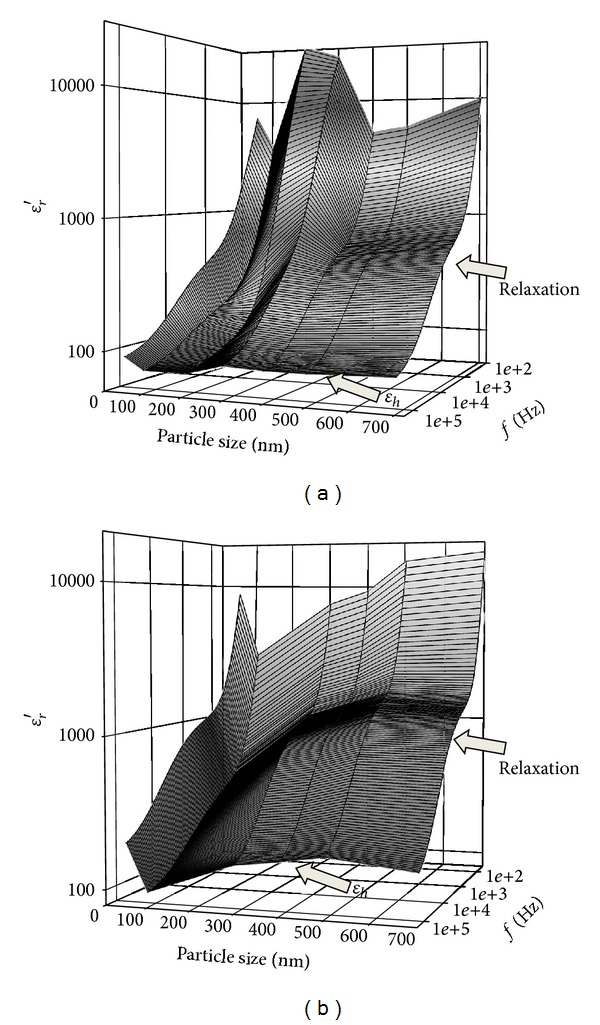
Three-dimensional representations of the nanosize dependence of the permittivity spectra of the BaTiO_3_ slurries with two solids loadings (a) 10 vol% and (b) 30 vol%.

Figure 1 shows the 3D representations of the particle size-dependent dielectric relaxation spectra of BaTiO_3_ powders dispersed in propylene carbonate (PC) with 10 vol% and 30 vol% solids loadings, respectively. A clear relaxation behavior can be seen at near 10^4^ Hz, which is aroused from the interfacial polarization between the BaTiO_3_ powders and the PC. By using Bruggeman-Hanai equation [9]

(1) $$\begin{array}{c} \frac{\varepsilon_{h}-\varepsilon_{p}}{\varepsilon_{m}-\varepsilon_{p}}{\left(\frac{\varepsilon_{m}}{\varepsilon_{h}}\right)}^{1/3}=1-\varphi , \end{array}$$

the permittivity of BaTiO_3_ powders (*ε*_*p*_) can be calculated directly from the high-frequency limiting permittivity of the slurry (*ε*_*h*_) in the case that the volume fraction of the powders in the slurry (*φ*) and the permittivity of the PC  (*ε*_*m*_) are known accurately. So *ε*_*h*_ is a significant dielectric parameter in order to obtain the *ε*_*p*_ with small margin of error. 

**Figure 2:**
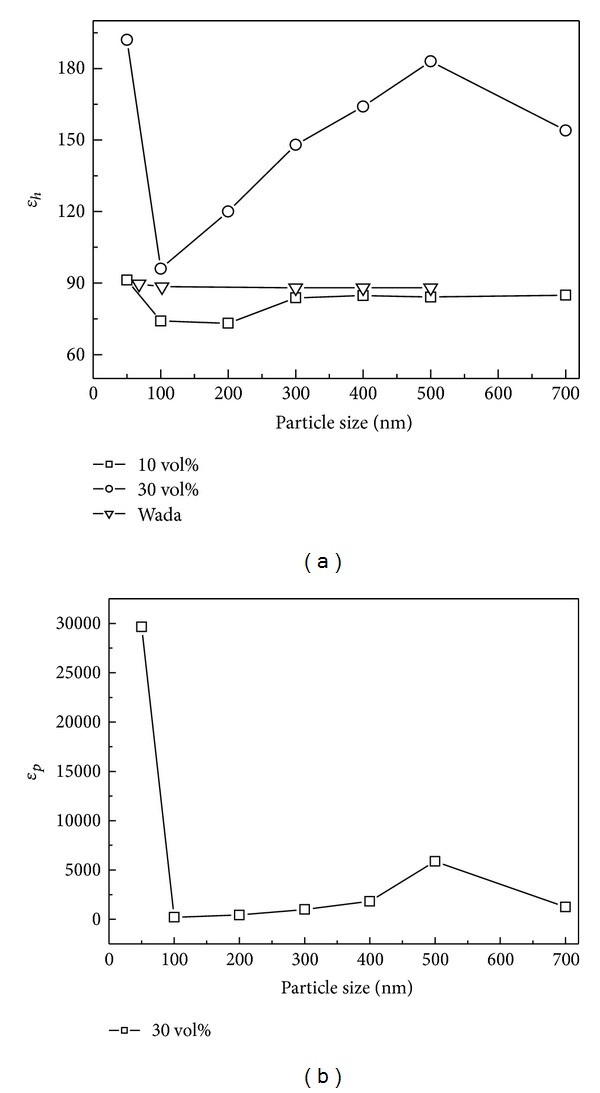
The size dependence of the relative dielectric parameters of the BaTiO_3_ slurries.

Figure 2(a) shows the particle size dependence of *ε*_*h*_ of BaTiO_3_ slurries with different *φ*. According to the nonlinear relationship between *ε*_*h*_ and *φ* in (1), the *ε*_*h*_ will increase with increasing *φ* if the *ε*_*p*_ is larger than the *ε*_*m*_. The permittivity of the PC is about 67 at room temperature, which is much smaller than the permittivity of BaTiO_3_ powders (1000~5000). So it is reasonable that the *ε*_*h*_ of 30 vol% BaTiO_3_ slurry is much higher than the one of  10 vol% slurry at each particle size. Furthermore, the BaTiO_3_ slurries with different volume fractions display distinct particle size dependence. For the slurry with 10 vol% solids loading, *ε*_*h*_ keeps constant basically on the size range from 700 nm to 300 nm, which agrees with the results in Wada's research [8]. For the slurry with 30 vol% solids loading, however, the *ε*_*h*_ displays a nonlinear size dependence similar with the results from solids method [10]. The difference implies that the analysis of the solids loading might explain the discrepancy between the previous results from the slurry and pellet methods.

Now, the permittivity of the BaTiO_3_ powders is supposed to decrease from 4000 to 3000 with decreasing particle size, and the permittivity of the BTiO_3_ slurries with different given solids loading is calculated by (1). According to the results listed in Table 1,  *φ* dependence of change rate in *ε*_*h*_(Δ*ε*_*h*_% = (*ε*_*h*,2_ − *ε*_*h*,1_)/*ε*_*h*,2_ × 100%) can be found. With the volume fraction becoming diluted from 25% to 4%, the Δ*ε*_*h*_% decreases gradually from 1.90% to 0.20%. It means that the diluter the slurry is, the higher the requirement to the measurement accuracy will be. In experimental measurements, there are always both systematic and random errors, which are mixed into the original data usually. For the dilute slurry, the small Δ*ε*_*h*_% is more inclined to be interfered by the systematic and random errors and offers inaccurate information. So the solids loading of the slurry should be increased high enough in order to weaken the confused influence of the systemic error on the original data. 

Considering the positive effect of higher solids loading on the accuracy of the slurry method, the *ε*_*h*_ from Figure 1(b) was assigned into (1) for calculating the permittivity of BaTiO_3_ powders, *ε*_*p*_.  Figure 2(b) shows the size dependence of *ε*_*p*_, which has the similar size dependence with the corresponding *ε*_*h*_. It can be found that the *ε*_*p*_ increased obviously with the particle size decreasing from 700 nm to 500 nm, and then decreased gradually with the particle size decreasing from 500 nm to 100 nm. The result agrees with the current postulation from the pellet method. The *ε*_*p*_ dependence in fine BaTiO_3_ structures is driven by a combined effect of the ferroelectric core whose nonlinearity is gradually reducing, together with the increasing amount of the nonferroelectric “dead-layer” grain boundaries, when reducing the grain size down to tenths of nanometers [1]. However, the *ε*_*p*_ with 50 nm particle size appeared with significant high value, which was reported only in Wada's slurries method. There is not enough attention for explaining the phenomenon yet, and we will focus on it in our following research.

## 4. Conclusions

 The importance of the solids loading in the accuracy of the sully method was researched in order to get the accurate nanosize effect of BaTiO_3_ powders. By means of comparing the dielectric data and calculation of the BaTiO_3_ slurries with two different solids loadings, 10 vol% and 30 vol%, the higher one was found to possess lower calculation error. Based on the finding, the conflicting nanosize dependence of the BaTiO_3_ powders obtained from previous slurry and pellet method was attributed to the low solid loading used in the slurry method. 

## Figures and Tables

**Table 1:** The response sensitivity of *ε*_*h*_ to the *ε*_*p*_ under different solids loadings.

Given φ	ε_h,1_	*ε*_*h*,2_	Δ*ε*_*h*_%
	(ε_p_ = 3000)	(*ε*_*p*_ = 4000)	
4%	74.547	74.695	0.20
10%	89.127	89.622	0.56
17%	111.08	112.29	1.09
25%	145.27	148.03	1.90
